# Coupled Nanomechanical Graphene Resonators: A Promising Platform for Scalable NEMS Networks

**DOI:** 10.3390/mi14112103

**Published:** 2023-11-16

**Authors:** Brittany Carter, Uriel F. Hernandez, David J. Miller, Andrew Blaikie, Viva R. Horowitz, Benjamín J. Alemán

**Affiliations:** 1 Department of Physics, University of Oregon, Eugene, OR 97403, USAablaikie103@gmail.com (A.B.); 2 Physics Department, Hamilton College, Clinton, NY 13323, USA; vhorowit@hamilton.edu; 3 Materials Science Institute, University of Oregon, Eugene, OR 97403, USA; 4 Center for Optical, Molecular, and Quantum Science, University of Oregon, Eugene, OR 97403, USA; 5 Phil and Penny Knight Campus for Accelerating Scientific Impact, University of Oregon, Eugene, OR 97403, USA

**Keywords:** graphene, nanoelectromechanical systems (NEMS), coupling, resonator networks

## Abstract

Arrays of coupled nanoelectromechanical resonators are a promising foundation for implementing large-scale network applications, such as mechanical-based information processing and computing, but their practical realization remains an outstanding challenge. In this work, we demonstrate a scalable platform of suspended graphene resonators, such that neighboring resonators are persistently coupled mechanically. We provide evidence of strong coupling between neighboring resonators using two different tuning methods. Additionally, we provide evidence of inter-resonator coupling of higher-order modes, demonstrating the rich dynamics that can be accessed with this platform. Our results establish this platform as a viable option for realizing large-scale programmable networks, enabling applications such as phononic circuits, tunable waveguides, and reconfigurable metamaterials.

## 1. Introduction

Networks of coupled NEMS resonators have attracted recent attention for the promise of mechanical computing [1,2,3] applications and for the study of fundamental physics, including metamaterial [4,5,6,7] and collective dynamics [8,9]. To continue to scale the size and prospects of coupled NEMS resonator networks, we need to develop robust platforms that host strong coupling and are scalable in size and tunability. Suspended graphene resonators offer many properties [10,11] that could be essential for achieving large 2D tunable arrays [12], such as intrinsic nonlinearities [13] that enable network dynamics [6,14,15,16] and multiple forms of energy transduction [17] for tuning options [18,19]. Persistent phototuning [19] has also recently been demonstrated, opening the possibility for scalable tuning of large-scale graphene networks.

Many coupling schemes have been established to host tunable strong coupling between resonators by means of parametric [20,21] and electromechanical [22,23] coupling. However, these methods are limited in scalability because they are not persistent and often require individually addressing each resonator, which limits the dimension of scalability in these platforms. One coupling means that is persistent and scalable in 2D is direct mechanical strain coupling though a shared clamping point [24,25,26], bridge [9], or substrate [22]. Mechanical strain coupling has been demonstrated between spatially distinct graphene nanoribbons [27,28] but has been limited to a 1D linear chain. However, direct strain coupling has been utilized to construct a 2D network of coupled pillars [29], hinting that strain coupling may enable other material platforms to scale in 2D as well.

In this work, we present a 2D platform [16] that hosts persistent strain coupling between suspended graphene resonators. We show evidence of strong coupling between two and three resonators of varying sizes and locations, establishing the viability of the platform for diverse array-based resonator applications. Additionally, this platform accommodates rich dynamics with inter-resonator coupling of higher-order modes, which we observed between the second-order mode of a driven resonator and the fundamental mode of its neighbor. With this platform, we establish a means in which the unique properties of 2D suspended graphene resonators can be accessed to enable a broad range of large-scale network applications.

## 2. Materials and Methods

**Figure 1:**
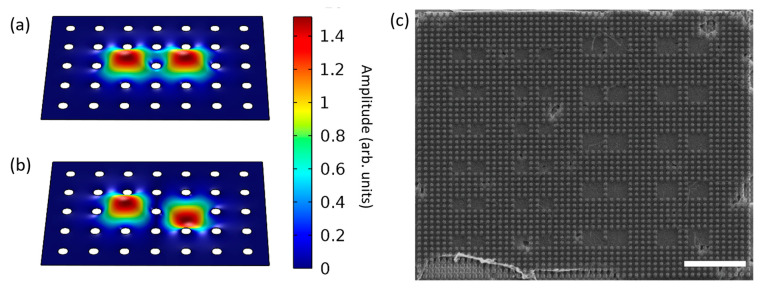
FEA simulations of (**a**) symmetric and (**b**) antisymmetric coupled resonator modes for pillar radius of 0.5 μm and pitch of 3 μm; (**c**) SEM of suspended graphene resonators with pillar pitch of 1 μm and radius of 0.25 μm. Scale bar is 10 μm.

To optimize the strain coupling between neighboring suspended graphene resonators, we designed a pillar array substrate [16,30,31,32] as the base structure for the network. The shared membrane between pillars would provide a mechanism for mechanical strain coupling between spatially distinct resonators. We selected pillar parameters that would lead to strong coupling by using finite element analysis (FEA) to determine the eigenfrequencies of the symmetric (Figure 1a) and antisymmetric (Figure 1b) modes of resonator pairs. We fabricated the platform by patterning Si/SiO_2_ substrates with pillar arrays and using a wet transfer method to suspend the graphene. Throughout the arrays, we intermittently omitted specific pillars to create resonators of different sizes and resonator pairs; see Figure 1c. We were able to suspend the transferred graphene almost fully on the denser arrays, as in Figure 1c. However, the suspension yield varied across the sample (see Appendix A for additional FEA and fabrication details). 

In this work, we used an optical drive/probe method to thermally drive and measure the out-of-plane motion of the resonators. We use two methods of tuning to demonstrate the versatility of this platform. Our first tuning method was to thermally tension the driven resonator only by applying a power offset to the modulated drive laser. Our second tuning method was to electrically tension all resonators on the sample by applying a bias between the graphene and the Si substrate [17]. Our optical apparatus also enabled scanning interference microscopy [33] (SIM) with fast scanning mirrors to raster the probe and collect spatial images of local areas of amplitude and phase (see Appendix A for additional details).

## 3. Results

**Figure 2:**
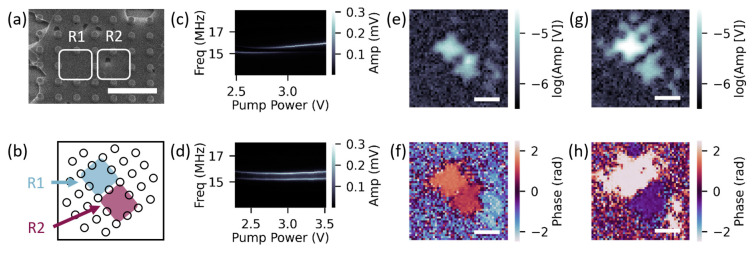
(**a**) SEM image of two neighboring coupled resonators, R1 and R2. Scale bar is 6 μm; (**b**) Dot array rotated at the same angle as SIM images. R1 labeled with blue shading and R2 labeled with maroon shading. Avoided crossing with R1 driven for (**c**) amplitude of R1 and (**d**) amplitude of R2. SIM images of (**e**) amplitude and (**f**) phase for R1 driven at $\omega_{-}/2\pi =15.45$ MHz. SIM images of (**g**) amplitude and (**h**) phase for R1 driven at $\omega_{+}/2\pi =16.21$ MHz. All SIM scale bars are 5 μm.

We first employed our thermal-tensioning tuning method to modulate the coupling strength between two neighboring resonators. With the drive and probe lasers aligned over the region highlighted as R1 in Figure 2a, we probed for coupling by measuring a spectrograph of the amplitude, shown in Figure 2c. In the resulting spectrograph, we observed an avoided crossing of a lower frequency mode, $\omega_{-}$, and a higher frequency mode, $\omega_{+}$, at a power offset of 2.8 V, implying the presence of coupling.

To confirm that the coupling was mechanical and to locate the coupled neighboring resonators, we took SIM images at both $\omega_{-}$ and $\omega_{+}$ with a drive power near the avoided crossing minimum to optimize each amplitude. For SIM measurements, we drove at a frequency slightly off resonance to avoid large heating fluctuations in amplitude and phase. The sample angle in the SIM images is depicted in Figure 2b. To compare the amplitude and phase of each active resonator, we analyzed a line cut of each SIM image through the center of each resonator. All reported uncertainty is calculated standard error from these linecuts. In the $\omega_{-}$ mode, we observed two distinct amplitude peaks, Figure 2e, that corresponded to the locations of R1 and R2, highlighted in Figure 2a. These two regions oscillated near in phase (R1 =1.47±0.03 rad and R2 =1.00±0.04 rad); Figure 2f, which is expected for the lower frequency mode of two coupled resonators, with slight differences likely due to heating fluctuations from scanning the probe laser. In the $\omega_{+}$ mode, we again observed two distinct amplitude peaks, Figure 2g, which corresponded to the same R1 and R2 locations. The two regions oscillated ~π out of phase (R1 =2.47±0.03 rad and R2 =−0.51±0.03 rad), Figure 2h, as expected for the higher frequency mode of two coupled resonators.

To verify the coupling between R1 and R2, we obtained a spectrograph of R2 by using the SIM image to reposition the probe laser over R2, while leaving the drive laser stationary to drive R1. In the resulting spectrograph, shown in Figure 2d, we again observed a lower and higher frequency mode that did not cross, with a minimum mode separation occurring at 2.9 V. This frequency was slightly higher than that for the R1 resonator, which may be due to heating and cooling effects associated with repositioning the probe laser. We also note that the avoided crossing curve shapes and amplitudes were less typical than observed in the R1 spectrograph, which may also be due to the probe repositioning. From the SIM spatial maps and the correlated avoided crossing curves, we conclude that the R1 and R2 resonators are coupled.

To determine the coupling strength, we calculated the minimum mode separation between $\omega_{-}$ and $\omega_{+}$ as

$$g=\omega_{+}-\omega_{-}=\Delta \omega$$

Based on the R1 avoided crossing spectrograph (Figure 2c), the coupling strength was g/2π≈400 kHz. Because this coupling strength exceeded the linewidths of the two modes (~150 kHz), this resonator pair was strongly coupled.

**Figure 3:**
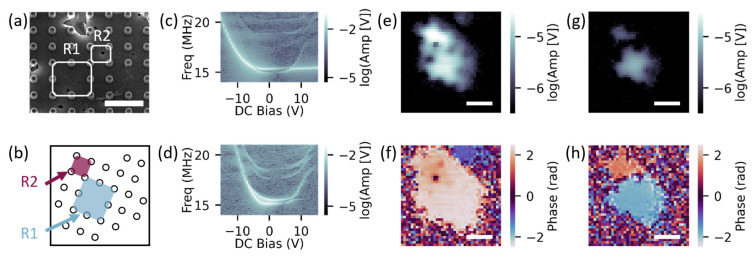
(**a**) SEM image of neighboring coupled resonators, R1 and R2. Scale bar is 6 μm; (**b**) Dot array rotated at the same angle as SIM images. R1 labeled with blue shading and R2 labeled with maroon shading. DC bias gate sweep avoided crossings with R1 driven for (**c**) amplitude of R1 and (**d**) amplitude of R2. SIM images of (**e**) amplitude and (**f**) phase for R1 driven at $\omega_{-}/2\pi =15.16$ MHz. SIM images of (**g**) amplitude and (**h**) phase for R1 driven at $\omega_{+}/2\pi =15.51$ MHz. All SIM scale bars are 5 μm.

We then used our electrical tuning method to characterize the coupling between an additional set of two resonators. We first positioned the drive and probe laser over R1, highlighted in Figure 3a, and probed for coupling by measuring an amplitude spectrograph. In the resulting spectrograph, shown in Figure 3c, we observed an avoided crossing of two modes, $\omega_{-}$ and $\omega_{+}$, at 6.4 V, implying that R1 was strongly coupled to at least one neighboring resonator. In this avoided crossing, we also observed applied bias ranges in which each mode tuned very little. This behavior is likely evidence of phototuning [19], or a change in the charge neutrality point of the suspended graphene due to a redistribution of charge [34]. 

To map the configuration of resonators coupled to R1, we took SIM images at $\omega_{-}$ and $\omega_{+}$ with zero applied voltage and an orientation as depicted in Figure 3b. In the $\omega_{-}$ mode, we observed two distinct amplitude peaks, Figure 3e, that corresponded to the regions, highlighted as R1 and R2 in Figure 3a,b. These two regions differed significantly in size ($Area_{R1}\approx 4\times Area_{R2}$). Because the frequency of the fundamental mode is inversely proportional to the width of the square membrane, it is possible that additional tensioning from the drive laser on R1 aligned the two individual resonance frequencies, resulting in the coupling of R1 and R2. In the $\omega_{-}$ mode, R1 and R2 oscillated near in phase (R1 =1.79±0.07 rad and R2 =2.26±0.02 rad); Figure 3f. In the $\omega_{+}$ mode, we observed two distinct regions of amplitude, Figure 3g, that corresponded to the same R1 and R2 regions and oscillated ~π out of phase (R1 =1.53±0.05 rad and R2 =−1.82±0.04 rad); Figure 3h. 

We confirmed the coupling between R1 and R2 by measuring a spectrograph of R2, Figure 3d, which revealed an avoided crossing between 6.4 V and 7.0 V. Based on the R1 avoided crossing curve, we calculated the coupling strength between R1 and R2 to be g/2π≈200 kHz. Considering estimated linewidths of ~120 MHz at the avoided crossing minimum, we conclude this resonator pair was strongly coupled.

**Figure 4:**
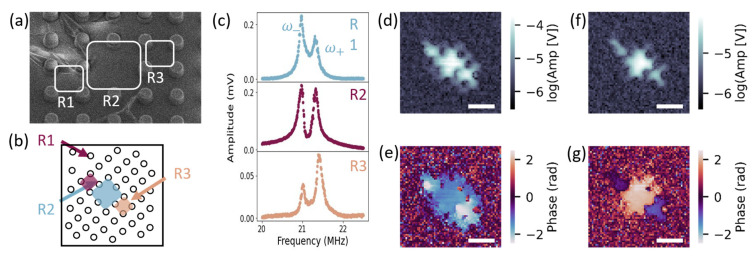
(**a**) SEM image of three coupled resonators, R1, R2, and R3. Scale bar is 5 μm; (**b**) Dot array rotated at the same angle as SIM images. R1 labeled with blue shading and R2 labeled with maroon shading; (**c**) Spectra of R1 (upper), R2 (middle), and R3 (lower). SIM images of (**d**) amplitude and (**e**) phase with R2 driven at $\omega_{1}/2\pi =20.99$ MHz. SIM images (**f**) amplitude and (**g**) phase with R2 driven at $\omega_{2}/2\pi =21.45$ MHz. All SIM scale bars are 5 μm.

Our pillar platform offers a high potential for 2D scalability, which we observed in the coupling between three adjacent resonators. We measured this coupling by aligning the drive and probe lasers over R2, highlighted in Figure 4a, and sweeping the drive frequency to locate resonance. In the resulting amplitude curve, shown in Figure 4c (middle), we observed two closely spaced but distinct peaks. 

To determine if these two peaks signified hybridized modes, we took SIM spatial images at $\omega_{-}$ and $\omega_{+}$ and with the orientation depicted in Figure 4b. In the $\omega_{-}$ mode, we observed three distinct amplitude peaks, Figure 4d, that all oscillated nearly in phase as seen in Figure 4e (R1 =0.23±0.13 rad, R2 =0.20±0.03 rad, and R3 =0.41±0.04 rad), implying coupling between a total of three resonators. Again, the observed oscillating regions, highlighted in Figure 4a,b, differed in size with the largest resonator, R2, subject to additional tensioning from the applied drive laser. In the $\omega_{+}$ mode, we again observed three distinct amplitude peaks, Figure 4f, in the same R1, R2, and R3 regions as $\omega_{-}$. In this mode, the two neighboring resonators, R1 and R3, oscillated out of phase with the driven R2 resonator (R1 =−0.23±0.17 rad, R2 =2.08±0.02 rad, and R3 =−0.62±0.18 rad), Figure 4g. Although we may expect that three strongly coupled resonators will exhibit three hybridized modes, when driving the middle resonator, it is possible that only two modes can be resolved (see Appendix B).

To confirm this coupling, we measured an amplitude spectrum of R1, shown in Figure 4c (upper), and R3, shown in Figure 4c (lower). In all three amplitude spectra, we observe two peaks at about the same $\omega_{-}$ and $\omega_{+}$ frequencies. We therefore conclude that this cluster of three resonators—R1, R2, and R3—are weakly to strongly coupled.

**Figure 5:**
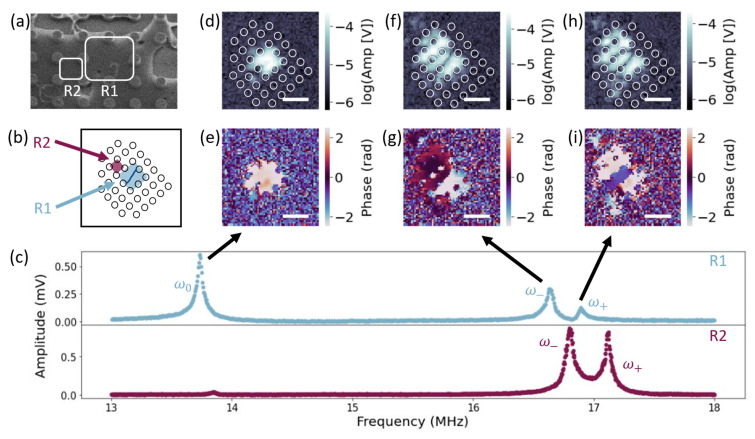
(**a**) SEM image of two coupled resonators, R1 and R2. Scale bar is 4.5 μm, (**b**) Dot array rotated at the same angle as SIM images. R1 labeled with blue shading and blue solid line to represent higher-order mode boundary. R2 labeled with maroon shading. (**c**) amplitude spectrum of R1 plotted with blue data points (upper) and amplitude of R2 plotted with maroon data points (lower). Amplitude peaks labeled as $\omega_{0}$, $\omega_{-}$, and $\omega_{+}$ in R1 spectrum. SIM images of (**d**) amplitude and (**e**) phase with R1 driven at $\omega_{0}/2\pi =13.85$ MHz. SIM images of (**f**) amplitude and (**g**) phase with R1 driven at $\omega_{-}/2\pi =16.74$ MHz. SIM images of (**h**) amplitude and (**i**) phase with R1 driven at $\omega_{+}/2\pi =16.97$ MHz. All SIM scale bars are 8 μm.

Our pillar platform offers unique 2D coupling dynamics between resonators including the inter-resonator coupling of higher-order modes. We detected higher-order mode coupling by measuring the amplitude spectrum of R1, highlighted in Figure 5a. In the spectrum, Figure 5c (upper), we measured a single peak $\omega_{0}$, close to 14 MHz, and two closely paced peaks, $\omega_{-}$ and $\omega_{+}$, near 17 MHz, implying that coupling may occur at a higher-order mode of R1. 

To further investigate this coupling, we took SIM images at drive frequencies of $\omega_{0}$, $\omega_{-}$, and $\omega_{+}$. Figure 5b depicts the angle of SIM imaging, which we also overlay on the SIM amplitude images, Figure 5d,f,h. In the $\omega_{0}$ mode, Figure 5d,e, we observed a region of high amplitude ($\sim 10^{-4}\;$V) with near constant phase, corresponding to the R1 region highlighted in Figure 5a,b. We interpret this mode to be the fundamental mode of R1. In the $\omega_{-}$ mode, we observed three distinct regions of amplitude, Figure 5f. Two of the peak amplitude regions occurred within R1, with mode boundary drawn as a solid line through the R1 resonator in Figure 5b. The phase between the two amplitude peaks within the R1 region, Figure 5g, differed by ~π (upper half =0.30±0.10 rad and lower half =−2.46±0.07 rad), as expected for the second-order mode of a 2D graphene drumhead resonator [33]. The third amplitude peak corresponded to the region highlighted as R2 in Figure 5a,b. R2 oscillated near in phase with the upper half of the R1 resonator (R2 =0.45±0.38 rad), Figure 5g. This phase pattern creates the least amount of curvature in the membrane for the case of coupling a fundamental mode to a second-order mode and is therefore expected to correspond to the lower energy state.

In the $\omega_{+}$ mode, we again observed three distinct amplitude peaks: Figure 5h. In this mode, R2 oscillated out of phase with the upper half of the R1 resonator and near in phase with the lower half (R2 =1.96±0.37 rad, upper half of R1 =−0.93±0.07 rad, and lower half of R1 =2.49±0.18 rad); Figure 5i. This phase pattern creates more curvature in the membrane and is therefore expected to result in a higher energy state. Higher-order mode coupling between these two resonators may be possible due to the differing sizes of R1 and R2. 

To confirm this coupling, we measured an amplitude spectrum of R2, shown in Figure 5c (lower). In this spectrum, we observed a small peak ($\sim 4\times 10^{-5}$ V) near the fundamental mode of R1 and two much larger peaks ($\sim 9\times 10^{-4}$ V) around $\omega_{-}$ and $\omega_{+}$, with slight shifts due to heating. Due to the amplitude difference, and the homogeneous phase behavior of R2 observed in both the $\omega_{-}$ and $\omega_{+}$ modes, we expect that the small peak near the fundamental mode of R1 does not correspond to a resonance of R2. We therefore conclude that the mode splitting observed around 17 MHz is due to coupling between the second-order mode of the driven resonator R1 and the fundamental mode of R2.

## 4. Discussion

We have demonstrated the tunability of our resonator platform by tensioning the resonators both thermally and electrically to collect avoided crossing spectrograph. Thermally driving the resonators often led to coupling between resonators asymmetric in size, which may be advantageous for tuning neighboring resonators at different rates under a universally applied back gate. This platform also has the potential for scalable phototuning [19], which would enable persistent and individual tensioning of suspended graphene regions without the need for individual back gates. Additionally, if the shared membrane between neighboring resonators were to be tensioned with any of the discussed methods, it may be possible to tune coupling strength between resonators.

The additional evidence of inter-resonator, higher-order mode coupling highlights the possibility of rich dynamics that are an asset unique to this graphene platform. Coupling between higher-order modes of spatially separate resonators can be utilized as means of turning coupling on and off between different areas of the network or to achieve coupling between resonators of different sizes without additional thermal tensioning.

Although we focused our analysis on strong coupling, the SIM spatial maps exhibit oscillating regions corresponding to areas of weak coupling. Weak coupling is an important network parameter, as it is essential for realizing many oscillator-based phenomena [8]. Although weak coupling is often difficult to measure [35], our SIM spatial imaging technique illuminates weakly coupled regions, enabling a more accurate model of the network.

## 5. Conclusions

In conclusion, we have presented evidence of coupling between two resonators, three resonators, and resonators of with a higher-order mode in a pillar-based graphene NEMS network platform. This platform thus enables persistent 2D mechanical strain coupling, with the unique properties of graphene offering potential for scalable tunability. With this platform, we can achieve large-scale arrays with persistent coupling for applications such as computing schemes [1,2,3], experimentation of tunable metamaterial [6,7,36,37], and physical simulation of natural and artificial networks [38,39]. 

## Figures and Tables

**Figure A1:**
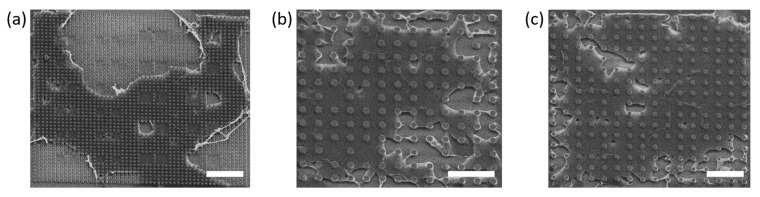
(**a**) Partial suspension of graphene over array with pillar radius of 0.25 μm and pillar pitch of 1 μm, (**b**) Suspended graphene over array with pillar radius of 0.75 μm and pillar pitch of 3.75 μm, (**c**) Suspended graphene over array with pillar radius of 0.5 μm and pillar pitch of 3 μm. All scalebars are 9 μm.

**Figure A2:**
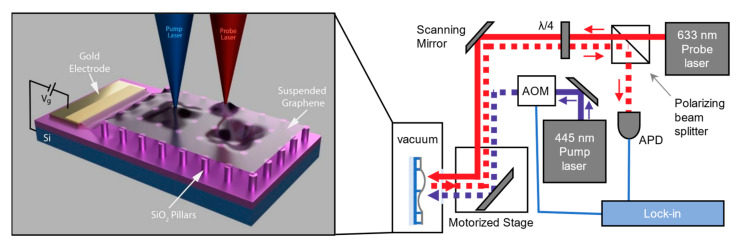
A schematic of the optical setup (**right**) used to drive and detect the graphene-pillar devices (**left**).

**Figure A3:**
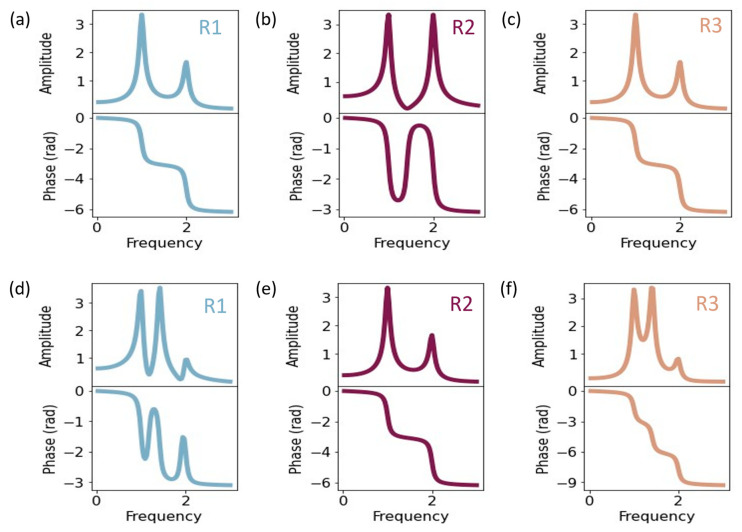
Simulated amplitude (upper) and phase (lower) spectra for three mass model for the case of the drive force applied to one of the edge masses for (**a**) driven edge mass, (**b**) middle mass, and (**c**) last edge mass. Simulated amplitude (upper) and phase (lower) spectra for three mass models for the case of the drive force applied to the middle mass for (**d**) first edge mass, (**e**) driven middle mass, and (**f**) last edge mass.

## Data Availability

The data that support the findings of this study are available from the corresponding author upon request.

## Appendix A. Fabrication, Simulation, and Measurement

We fabricated the suspended graphene resonator devices using standard nanofabrication processes to pattern the base substrate, followed by a wet transfer method to suspend the graphene. We also included a via electrode contacting the doped silicon substrate and a top electrode contacting the graphene, such that we could electrically bias the graphene to increase the tension.

We began our process with Si/SiO_2_ substrates with 1 μm of commercially grown wet oxide. We first patterned via electrodes, such that we could electrically contact the Si layer of the substrate from the top of the sample. We defined the via area with photolithography and etched 1 μm of SiO_2_ using a wet buffered oxide etch. We then deposited 10 nm/40 nm of Ti/Pt to contact the exposed silicon. We fabricated the pillars by using electron beam lithography to pattern dot arrays into PMMA A4 resist. After developing the dot patterns, we deposited a thin layer of chromium (~20 nm) to act as a negative mask for etching. To form the pillars, we etched ~600 nm of the exposed SiO_2_ with a CHF_3_ reactive ion etch (RIE). Finally, we deposited 10 nm/40 nm of Ti/Pt as a top electrode on the SiO_2_ using photolithography to define the pattern.

We suspended the graphene over the defined SiO_2_ features using a wet transfer method [32]. We began the transfer with commercially grown graphene on copper foil. We applied a layer of PMMA A11 resist to graphene/copper sheet to support the graphene layer during the transfer. We then dried the sheet in air for 10–15 min before carefully floating it copper side down in a solution of 40 mg/mL ammonium persulfate to etch the copper. Once the copper foil was completely etched, we transferred the PMMA/graphene sheet to float in three successive water baths to dilute any remaining ammonium persulfate. While the PMMA/graphene sheet was in the final water bath, we prepared the Si/SiO_2_ substrate with an O_2_ plasma to clean the surface and make it hydrophilic for better adhesion. We then used the substrate to scoop the PMMA/graphene sheet directly from the surface of the final water bath and dried it overnight in air to minimize any liquid trapped between the graphene and SiO_2_. We transferred graphene to arrays with a range of pillar radii, r, from 0.25–0.75 μm and pitches, a, from 1–3.75 μm. The sample was then baked for 5 min at 105 °C to soften the PMMA and allow the graphene to adhere better to the surface of the sample. We removed the PMMA layer by soaking the sample in Remover PG for 5–6 h. To minimize tensions in the graphene that occur when drying, we used a critical point dryer to transfer the sample from solution into air.

We used FEA to determine the eigenfrequencies of the symmetric and antisymmetric modes to predict the coupling strength between neighboring resonators using the condition [35]

$$Q\left(\frac{\Delta \omega }{2\omega_{0}}\right)>1$$

where Δω is the frequency difference between the first and second hybridized modes, $\omega_{0}$ is the average of the two frequencies, and Q is the quality factor. In this calculation, we conservatively estimate the quality factor to be Q=100 based on previously measured suspended graphene devices [19,33,40,41]. We selected the range of pillar radii and pitches for the arrays to be both spread out enough to host strong coupling and dense enough to allow for successful graphene suspension. Although overall we obtained higher yield on arrays with larger r/a ratios, we also observed certain areas of the sample transfer better than others. As an example, Figure A1a shows an array with the same dimensions as in Figure 1c but located in a difference area of the sample and only partially covered with suspended graphene. The largest pitch with suspended graphene devices was 3.75 μm, shown in Figure A1b, and the largest r/a ratio was 0.5/3, shown in Figure A1c.

To simulate the response of the proposed geometry, we used the membrane physics package (mem) in COMSOL. The material parameters included a membrane thickness of 0.335 nm, a Young’s modulus of 1.0149 TPa, a mass density of 6600 kg/m^3^, and a Poisson’s ratio of 0.17. We used a Linear Elastic Material with an initial strain of $-1\times 10^{-4}$ in the boundary. We set the initial values on the boundary for the displacement and velocity fields to zero. For a clamped edge configuration, we set the displacement of the geometry boundaries in x, y, and z directions to zero. For the mesh size, we used an “extremely fine”, physics-controlled mesh. The study manually searched for 10 eigenfrequencies around a MHz using the closest absolute value eigenfrequency search method. The MUMPS solver used a 1.2 memory allocation factor with a pivot threshold of 0.1.

We drove the suspended graphene resonators and read out the corresponding out-of-plane motion using an optical drive/probe method. A schematic of the optical setup and device geometry is provided in Figure A2. The drive was a 445 nm laser modulated with an acousto-optic modulator to thermally tension the graphene and drive out-of-plane motion. The probe was a 633 nm laser, in which the reflected interference signal was measured via lock-in detection to extract the corresponding amplitude and phase of an oscillating resonator. We modulated the coupling between resonator pairs with the 445 nm heating laser or by applying a gate bias $V_{g}$ between the top electrode and the back gate.

## Appendix B. Three Coupled Resonator Model

Typically, we expect three strongly coupled resonators to have three hybridized modes based on the eigenvectors of a linear chain of masses and springs. Therefore, because we only observed two modes in the spectra of the coupled system in Figure 5, it is possible that there is weak coupling present in the system, or mode splitting that is unresolvable in the spectra [35]. However, by modeling the three drumhead resonators as a linear chain of masses and springs with an applied drive force, we find that it is possible for only two hybridized modes to emerge, even when all three resonators are strongly coupled.

In our linear mass and spring model, each mass had an intrinsic spring and was connected to the nearest neighbor mass through a coupling spring. We set all masses and spring constants to be equal and we set the damping to be one order of magnitude less than the individual resonance frequencies $\omega_{0}=\sqrt{k/m}.$ We found that when the drive force was applied to one of the edge masses, the eigenvector behavior of three coupled masses emerged; three hybridized modes in which all masses oscillated in phase for the first mode, the outer masses oscillated in phase and the center mass was stationary for the second mode, and finally the outer masses oscillated in phase and the center mass oscillated out of phase for the third mode. Simulated amplitude and phase spectra are shown for the outer masses in Figure A3a,c and for the middle mass in Figure A3b.

With the same parameter values, we repeated this simulation for the drive force applied to the middle mass, as was the case for the coupled system in Figure 5. In this simulation, we observed only two hybridized modes, in which all masses oscillated in phase for the first mode, and the outer masses oscillated in phase while the middle mass oscillated out of phase for the second mode. Simulated amplitude and phase spectra are shown for the outer masses in Figure A3d,f and for the middle mass in Figure A3e. This matches the behavior observed in the three measured graphene drumhead resonators in Figure 5. We therefore conclude that although only two modes were detected, it is possible that the three measured graphene resonators were strongly coupled.
